# MCMDA: Matrix completion for MiRNA-disease association prediction

**DOI:** 10.18632/oncotarget.15061

**Published:** 2017-02-03

**Authors:** Jian-Qiang Li, Zhi-Hao Rong, Xing Chen, Gui-Ying Yan, Zhu-Hong You

**Affiliations:** ^1^ College of Computer Science and Software Engineering, Shenzhen University, Shenzhen, 518060, China; ^2^ School of Software, Beihang University, Beijing, 100191, China; ^3^ School of Information and Control Engineering, China University of Mining and Technology, Xuzhou, 221116, China; ^4^ Academy of Mathematics and Systems Science, Chinese Academy of Sciences, Beijing, 100190, China; ^5^ Xinjiang Technical Institute of Physics and Chemistry, Chinese Academy of Science, ÜRÜMQI, 830011, China

**Keywords:** miRNA, disease, miRNA-disease association, matrix completion

## Abstract

Nowadays, researchers have realized that microRNAs (miRNAs) are playing a significant role in many important biological processes and they are closely connected with various complex human diseases. However, since there are too many possible miRNA-disease associations to analyze, it remains difficult to predict the potential miRNAs related to human diseases without a systematic and effective method. In this study, we developed a Matrix Completion for MiRNA-Disease Association prediction model (MCMDA) based on the known miRNA-disease associations in HMDD database. MCMDA model utilized the matrix completion algorithm to update the adjacency matrix of known miRNA-disease associations and furthermore predict the potential associations. To evaluate the performance of MCMDA, we performed leave-one-out cross validation (LOOCV) and 5-fold cross validation to compare MCMDA with three previous classical computational models (RLSMDA, HDMP, and WBSMDA). As a result, MCMDA achieved AUCs of 0.8749 in global LOOCV, 0.7718 in local LOOCV and average AUC of 0.8767+/−0.0011 in 5-fold cross validation. Moreover, the prediction results associated with colon neoplasms, kidney neoplasms, lymphoma and prostate neoplasms were verified. As a consequence, 84%, 86%, 78% and 90% of the top 50 potential miRNAs for these four diseases were respectively confirmed by recent experimental discoveries. Therefore, MCMDA model is superior to the previous models in that it improves the prediction performance although it only depends on the known miRNA-disease associations.

## INTRODUCTION

MicroRNA (miRNA) is a kind of short non-coding single-stranded RNA (∼22nt) which can regulate the gene expression by binding to the 3′ untranslated regions (UTRs) of its target messenger RNA (mRNA) through base pairing [1, 2]. There are significant differences between the miRNAs in different tissues and different growth stages, which means that miRNAs have differential spatial and temporal expression patterns [3]. Based on plenty of biological experiments, researchers now believe that these small molecules have a wide range of regulation effects on eukaryotic gene expression, not only in human genes but also in genes of many other species [4]. Up to now, researchers have discovered that miRNAs are involved in a series of critical life processes, including early cell growth, proliferation, differentiation [5, 6], apoptosis, death [7], fat metabolism and so on. Therefore, it is no wonder that miRNAs are closely related to many complex human diseases [8, 9]. For example, studies have implicated that miRNA-143 and miRNA-145 are constantly down-regulated in colorectal tumors [10] and recently Croce et al. also have shown that the downregulation of these miRNAs is a common occurrence in breast carcinomas [11]. Besides, studies by Takamizawa et al. [12] and Yanaihara et al. [13] have presented evidence that transcripts of certain let-7 homologs are significantly downregulated in human lung cancer. Based on real-time polymerase chain reaction (PCR), the analysis of miRNA arrays using pooled RNA samples from five gastric cancer patients indicates that the expression of miRNA-107, miRNA-21, miRNA-196a, miRNA-26b, miRNA-9, miRNA-142-3p, miRNA-30b, miRNA-150, miRNA-191, and miRNA-17 was found to be upregulated [14]. However, it is expensive and time-consuming to identify the associations between miRNAs and diseases using experimental methods. Considering that large numbers of miRNA-associated datasets are available, computational methods are efficient in predicting miRNA-disease associations in that they can select the most promising associated miRNAs for further experimental studies [15–17]. Therefore, it is necessary for us to make further efforts and develop efficient computational models to predict the potential miRNA-disease associations [16, 18–31].

Many computational methods have been established to predict the potential associations between miRNAs and diseases depending on the assumption that miRNAs with similar functions are more likely to have connections with diseases which share similar phenotypes [32, 33]. Jiang et al. [34] proposed a hypergeometric distribution-based model to predict miRNA-disease associations based on disease phenotype similarity network, miRNA functional similarity network, and known human disease-miRNA association network. However, this method strongly depends on the miRNA-target interactions with a high rate of false positive and false negative samples. Moreover, Shi et al. [35] presented a new model by implementing random walk algorithm on protein-protein interaction (PPI) network based on the idea that miRNAs whose target genes are related to certain diseases are more likely to be associated with these diseases. They made use of the miRNA–target interactions, disease–gene associations, and PPIs to acquire potential associations between the miRNAs and diseases. Mork et al. [36] proposed a miRPD method with the help of protein-disease interactions as well as protein-miRNA interactions, where not only disease-related miRNAs but also potential disease-related proteins were analyzed. By integrating known disease–gene associations and miRNA-target interactions, Xu et al. [37] introduced a miRNA prioritization method which need not rely on the known miRNA-disease associations. Instead, what they needed to do was to evaluate the similarity between the targets of miRNAs and disease genes. Nevertheless, all the methods mentioned above suffered from the miRNA-target interactions with high false positive and false negative samples, which could significantly reduce the accuracy of the aforementioned models.

Researchers also proposed some other computational models without relying on miRNA-target interactions. Based on miRNA functional similarity, disease semantic similarity, disease phenotype similarity, and miRNA-disease associations, Xuan et al. [38] presented an HDMP model which analyzed the miRNAs related to the diseases by considering the functional similarities of the miRNA’s k most similar neighbors. Compared with the previous methods, HDMP assigned higher weight to the miRNAs in the cluster and family since they are more likely to be associated with similar diseases. When applied to new diseases without some known related miRNAs, however, HDMP is unable to work since it strongly depends on the neighbors of the miRNAs. Besides, HDMP is based on a local similarity measure rather than a global measure which can notably promote the prediction performance. Xuan et al. [39] introduced another model called MIDP based on random walk, which exploited the characteristics of the nodes and the various ranges of topologies. The labeled nodes in MIDP were assigned higher transition weight than the unlabeled nodes, which efficiently exploited the prior information of nodes and various ranges of topologies. What is worth mentioning is that MIDP effectively relieved the negative effect of noisy data. MIDP also extended the walk on a miRNA-disease bilayer network to predict candidate specially for the diseases without any known miRNAs. Recently, Zeng et al. [40] utilized matrix completion to predict the miRNA-disease associations based on miRNA-miRNA network and disease-disease network. The method contributed multiple feature sets to address problems related to insufficient miRNA-disease association data. The method could be applied to predict unknown miRNA-disease associations and new pathogenic miRNAs for well-characterized diseases. Chen et al. [41] proposed RWRMDA model which integrated miRNA-miRNA functional similarity and known miRNA-disease associations information to predict miRNA-disease associations. RWRMDA was motivated based on the investigation that global similarity measures are better in predicting the associations between miRNAs and diseases than the previous local network similarity measures. Still, this method fails to predict miRNAs associated with new diseases without any known related miRNAs. Chen et al. [16] presented another model called WBSMDA based on miRNA functional similarity, disease semantic similarity, miRNA-disease associations, and Gaussian interaction profile kernel similarity for miRNAs and diseases. WBSMDA makes a breakthrough in that it succeeds in predicting related miRNAs for new diseases without known related miRNAs and new miRNAs without known related diseases. Recently, Chen et al. [42] presented a model of HGIMDA using miRNA functional similarity, disease semantic similarity, miRNA-disease associations, and Gaussian interaction profile kernel similarities. In HGIMDA, the new miRNA functional similarity network was obtained by combining miRNA functional similarity network with Gaussian interaction profile kernel similarities for miRNAs. The process of calculating new disease similarity network was quite similar. Then, a heterogeneous graph was obtained by combining new miRNA functional similarity network, new disease similarity network and known miRNA-disease associations. Moreover, the potential association between a disease and a miRNA could be inferred based on an iterative equation if they didn’t have known association. It has been verified that HGIMDA obtained a high prediction performance.

In addition, several computational models have considered machine learning methods. For instance, Xu et al. [43] developed a miRNA target-dysregulated network (MTDN) based on miRNA-target interactions as well as miRNA and mRNA expression profiles. Besides, MTDN implemented support vector machine (SVM) classifier to distinguish positive miRNA-disease associations from negative ones. Nevertheless, it is still fairly difficult to obtain the negative miRNA-disease associations today, which seriously decreases the prediction performance of this computational model. Chen et al. [15] presented a RLSMDA model based on semi-supervised learning which calculated the semantic similarity between different diseases. It is worth mentioning that RLSMDA could identify related miRNAs for diseases without any known associated miRNAs, meanwhile avoiding the problem of using negative associations between miRNAs and diseases. The trouble of RLSMDA is how to find the appropriate parameters and how to combine the classifiers from miRNA space and disease space together. Chen et al. [19] developed another computational model called RBMMMDA based on miRNA-disease associations which presented restricted Boltzmann machine (RBM) which is a two-layer undirected graphical model consisting of layers of visible and hidden units. Compared to the previous models, RBMMMDA could obtain not only new miRNA-disease associations but also corresponding association types. However, it is still too difficult to learn the complex parameters.

In this study, we developed an effective computational model of Matrix Completion for MiRNA-Disease Association prediction model (MCMDA) using matrix completion algorithm based on the known miRNA-disease associations to predict the potential miRNA-disease associations. Compared to the previous computational models, MCMDA predicts the miRNA-disease associations by using the matrix completion algorithm, which is of high efficiency to update the low-rank miRNA-disease matrix. Besides, negative associations which are required in some previous computational models are not needed in MCMDA. To evaluate the effectiveness of MCMDA, global and local LOOCV as well as 5-fold cross validation were introduced. The AUCs of global and local LOOCV were respectively 0.8749 and 0.7718, and the model obtained the average AUC of 0.8767+/−0.0011 on 5-fold cross validation. Besides, the top 10 and top 50 miRNAs related to colon neoplasms, kidney neoplasms, lymphoma and prostate neoplasms obtained by MCMDA were examined in dbDEMC [44] and miR2Disease [45] database. As a result, 84%, 86%, 78% and 90% of the top 50 potential miRNAs for these four complex diseases were respectively confirmed by recent experimental discoveries. Thus, it proves that MCMDA is effective in predicting potential miRNA-disease associations and it has significant advantages over the previous methods although MCMDA only depends on known miRNA-disease associations.

## RESULTS

### Performance evaluation

We used global and local LOOCV as well as 5-fold cross validation based on the known miRNA-disease associations in HMDD database to evaluate the performance of MCMDA. Meanwhile, MCMDA were compared with three previous classical computational methods: WBSMDA [16], RLSMDA [15] and HDMP [38]. In LOOCV evaluation, each known association in the database was regarded as the test sample in turn while the other known associations were regarded as training samples. The miRNA-diseases without known association evidences were considered as candidate samples. The scores of all miRNA-disease pairs could be obtained after MCMDA was implemented. In global LOOCV, the score of the test sample was compared with the scores of all the candidate samples while in local LOOCV, the test sample was merely compared with the scores of the candidate samples which included the particular disease in the test sample. In 5-fold cross validation, the known miRNA-disease associations were randomly divided into five disjoint parts. Each time, one part was picked out as test samples and the other four parts were treated as training samples. Still, the miRNA-disease pairs without known association evidences were regarded as candidate samples. Then, the score of each test sample were compared with the scores of all the candidate samples, respectively. This procedure was repeated five times until each known association was used as test sample and its score was compared with the scores of the candidate samples. Those test samples whose ranks exceeded the given threshold were considered to predict the miRNA-disease associations correctly.

**Figure 1:**
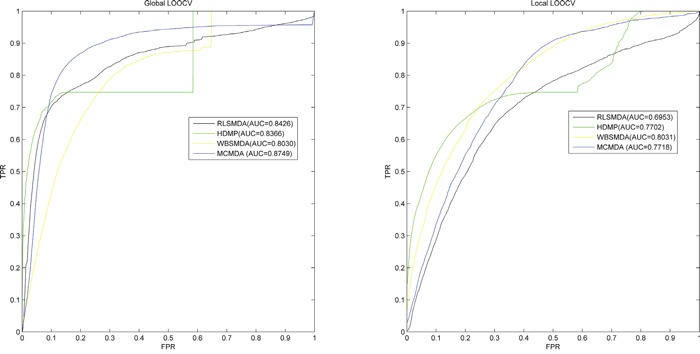
Performance evaluation comparison between MCMDA and three previous prediction models (RLSMDA, HDMP, WBSMDA) in terms of ROC curve and AUC based on global LOOCV and local LOOCV tested by known miRNA-disease associations in the HMDD database MCMDA achieved AUC of 0.8749 in global LOOCV and 0.7718 in local LOOCV. Thus, the performance of MCMDA is almost better than all the previous models in some degree and it proves to be effective in predicting the potential miRNA-disease associations.

Finally, we drew a receiver operating characteristics curve (ROC) to compare MCMDA with all the previous methods. In this curve, the true positive rate (TPR, sensitivity) and false positive rate (FPR, 1-specificity) were plotted [46]. Sensitivity represents the percentage of miRNA-disease test samples whose ranks exceeded the given threshold while specificity represents the percentage of negative miRNA-disease associations whose ranks were lower than the threshold [47]. The area under the ROC curve (AUC) was calculated to evaluate the accuracy of MCMDA. If AUC=1, MCMDA proves to be a prefect performance. AUC of 0.5 means that the method merely has a random prediction performance. As a result, the AUCs of MCMDA, WBSMDA, RLSMDA and HDMP were 0.8749, 0.8030, 0.8426, and 0.8366, respectively in global LOOCV. For local LOOCV, MCMDA, WBSMDA, RLSMDA and HDMP acquired AUCs of 0.7718, 0.8030, 0.8031 and 0.6953, respectively. The average AUCs of MCMDA, WBSMDA, RLSMDA, HDMP were 0.8767/−0.0011, 0.8185/−0.0009, 0.8569/−0.0020 and 0.8342+/−0.0010, respectively in 5-fold cross validation (See Figure 1). All in all, MCMDA turns out to be more effective in predicting potential miRNA-disease associations compared with the previous methods, especially considering that MCMDA merely depends on the known miRNA-disease associations in the database.

### Case studies

Furthermore, case studies of four significant diseases related to human health were implemented to practically evaluate the prediction accuracy of MCMDA. The top 10 and top 50 predicted miRNAs related with these diseases were examined by another two miRNA-disease databases, dbDEMC [44] and miR2Disease [45].

Colon Neoplasms is a malignant cancer which is commonly found in the boundary of rectum and sigmoid colon [48]. It is the third most common cancer and the third leading cause of cancer death for both men and women in the United States [49]. However, early patients of colon neoplasms only suffer from subtle symptoms [50], making the disease difficult to be detected. To make things worse, it is reported that its occurrence rate has an increasing trend these years [51]. Thus, it is urgent to predict the potential miRNAs related to colon neoplasms. With the help of the modern iatrology, many miRNAs have been confirmed to be correlated with colon neoplasms. For instance, miRNA-145 targets the insulin receptor substrate-1 and thus inhibits the growth of colon cancer cells [52]. Besides, miRNA-126, which is frequently lost in colon neoplasms cells, has the function of suppressing the growth of neoplastic cells by targeting phosphatidylinositol 3-kinase signaling [53]. MCMDA was implemented to predict the top 50 miRNAs associated with colon neoplasms. Therefore, 9 of the top 10 and 42 of the top 50 predicted miRNAs associated with colon neoplasms were verified by dbDEMC and miR2Disease database (See Table 1).

**Table 1:** Prediction of the top 50 predicted miRNAs associated with colon neoplasms based on known associations in HMDD database

miRNA	Evidence	miRNA	Evidence
hsa-mir-146a	dbdemc	hsa-mir-196a	dbdemc;miR2Disease
hsa-mir-155	dbdemc;miR2Disease	hsa-mir-29c	dbdemc
hsa-mir-122	unconfirmed	hsa-mir-223	dbdemc;miR2Disease
hsa-mir-21	dbdemc;miR2Disease	hsa-mir-143	dbdemc;miR2Disease
hsa-mir-34a	dbdemc;miR2Disease	hsa-let-7a	unconfirmed
hsa-mir-221	dbdemc;miR2Disease	hsa-mir-195	dbdemc;miR2Disease
hsa-mir-16	dbdemc	hsa-mir-200b	dbdemc
hsa-mir-125b	dbdemc	hsa-mir-214	dbdemc
hsa-mir-29a	dbdemc;miR2Disease	hsa-mir-106b	dbdemc;miR2Disease
hsa-mir-29b	dbdemc;miR2Disease	hsa-mir-23a	miR2Disease
hsa-mir-15a	dbdemc	hsa-mir-142	unconfirmed
hsa-mir-133a	dbdemc;miR2Disease	hsa-mir-31	dbdemc;miR2Disease
hsa-mir-222	dbdemc	hsa-mir-34c	miR2Disease
hsa-mir-20a	dbdemc;miR2Disease	hsa-mir-141	dbdemc;miR2Disease
hsa-mir-199a	unconfirmed	hsa-mir-148a	dbdemc
hsa-mir-26a	dbdemc;miR2Disease	hsa-mir-182	dbdemc;miR2Disease
hsa-mir-1	dbdemc;miR2Disease	hsa-mir-200a	unconfirmed
hsa-mir-19b	dbdemc;miR2Disease	hsa-let-7c	dbdemc
hsa-mir-19a	dbdemc;miR2Disease	hsa-mir-101	unconfirmed
hsa-mir-15b	miR2Disease	hsa-mir-192	dbdemc;miR2Disease
hsa-mir-18a	miR2Disease	hsa-mir-181a	dbdemc;miR2Disease
hsa-mir-92a	dbdemc	hsa-mir-9	dbdemc;miR2Disease
hsa-mir-206	unconfirmed	hsa-mir-133b	dbdemc;miR2Disease
hsa-mir-30b	dbdemc;miR2Disease	hsa-mir-34b	dbdemc;miR2Disease
hsa-mir-150	dbdemc;miR2Disease	hsa-mir-183	dbdemc;miR2Disease

The first column records top 1-25 related miRNAs. The second column records the top 26-50 related miRNAs.

Kidney neoplasms, also known as renal cancer, is a cancer starting in the cells of kidney that includes many different types [54]. The two most common types of kidney cancer are renal cell carcinoma (RCC) and transitional cell carcinoma (TCC, also known as urothelial cell carcinoma) of the renal pelvis [55]. The most common symptoms of kidney neoplasms patients are pains in the lumbar and hematuria [56]. Many existing kidney neoplasm-related miRNAs have been reported based on recent biological experiments. For example, the common target ACVR2B of five miRNAs (miRNA-192, miRNA-194, miRNA-215, miRNA-200c and miRNA-141) is strongly expressed in renal childhood neoplasms [57]. In addition, miRNA-23b, by targeting proline oxidase, a novel tumor suppressor protein, could function as an oncogene in renal cancer [58]. Thus, the decreasing miRNA-23b expression may prove to be an effective way of inhibiting kidney tumor growth [58]. Based on MCMDA, 7 of the top 10 potential miRNAs associated with kidney neoplasms were confirmed by deDEMC and miR2Disease database while 43 were verified of the top 50 (See Table 2).

**Table 2:** Prediction of the top 50 predicted miRNAs associated with kidney neoplasms based on known associations in HMDD database

miRNA	Evidence	miRNA	Evidence
hsa-mir-155	dbdemc	hsa-mir-92a	unconfirmed
hsa-mir-146a	dbdemc	hsa-mir-195	dbdemc
hsa-mir-122	dbdemc;miR2Disease	hsa-mir-126	dbdemc;miR2Disease
hsa-mir-34a	dbdemc	hsa-mir-29c	dbdemc;miR2Disease
hsa-mir-221	unconfirmed	hsa-mir-23a	dbdemc
hsa-mir-16	dbdemc	hsa-mir-143	dbdemc
hsa-mir-125b	unconfirmed	hsa-mir-223	dbdemc
hsa-mir-29a	dbdemc;miR2Disease	hsa-mir-214	dbdemc;miR2Disease
hsa-mir-133a	unconfirmed	hsa-let-7a	dbdemc
hsa-mir-29b	dbdemc;miR2Disease	hsa-mir-148a	dbdemc
hsa-mir-145	dbdemc	hsa-mir-200b	dbdemc;miR2Disease
hsa-mir-26a	dbdemc;miR2Disease	hsa-mir-31	dbdemc
hsa-mir-199a	dbdemc;miR2Disease	hsa-mir-142	unconfirmed
hsa-mir-222	dbdemc	hsa-mir-106b	dbdemc;miR2Disease
hsa-mir-1	dbdemc	hsa-mir-34c	dbdemc
hsa-mir-15b	dbdemc	hsa-mir-182	dbdemc;miR2Disease
hsa-mir-20a	dbdemc;miR2Disease	hsa-mir-200a	dbdemc
hsa-mir-17	dbdemc;miR2Disease	hsa-mir-101	dbdemc;miR2Disease
hsa-mir-30b	dbdemc	hsa-let-7c	dbdemc
hsa-mir-206	dbdemc	hsa-mir-181a	dbdemc
hsa-mir-19a	dbdemc	hsa-mir-9	dbdemc
hsa-mir-196a	dbdemc	hsa-mir-34b	dbdemc
hsa-mir-19b	dbdemc;miR2Disease	hsa-mir-183	dbdemc
hsa-mir-18a	dbdemc	hsa-mir-133b	unconfirmed
hsa-mir-150	dbdemc;miR2Disease	hsa-let-7b	unconfirmed

The first column records top 1-25 related miRNAs. The second column records the top 26-50 related miRNAs.

Lymphoma is a malignant tumor originating in the lymphatic hematopoietic system [59] which consists of two categories: non-Hodgkinlymphoma (NHL) and Hodgkin'slymphoma (HL) [60]. Lymphoma is thought to be associated with gene mutations, as well as viruses, pathogens, radiation, chemical drugs, autoimmune diseases, etc. [61]. For example, re-expression of miRNA-150 induces EBV-positive Burkitt lymphoma differentiation by modulating c-Myb *in vitro* [62]. Besides, the expressions of miRNA-21 and miRNA-210 in plasma of previously untreated lymphoma patient group were higher than those of the patients treated for 6 or more courses [63]. MCMDA model predicts the top 10 and top 50 miRNAs related to lymphoma. As a result, 9 of the top 10 and 39 of the top 50 potential miRNAs were confirmed in the deDEMC and miR2Disease database (See Table 3).

**Table 3:** Prediction of the top 50 predicted miRNAs associated with lymphoma based on known associations in HMDD database

miRNA	Evidence	miRNA	Evidence
hsa-mir-30b	dbdemc	hsa-mir-208a	unconfirmed
hsa-mir-148a	dbdemc	hsa-mir-26b	dbdemc
hsa-mir-373	dbdemc	hsa-mir-143	unconfirmed
hsa-mir-196a	dbdemc	hsa-mir-9	dbdemc
hsa-mir-23a	dbdemc	hsa-let-7b	dbdemc
hsa-mir-206	dbdemc	hsa-mir-96	dbdemc
hsa-mir-195	dbdemc	hsa-let-7d	dbdemc
hsa-mir-372	unconfirmed	hsa-mir-93	dbdemc
hsa-mir-199a	dbdemc	hsa-mir-483	unconfirmed
hsa-mir-15b	dbdemc	hsa-mir-371a	unconfirmed
hsa-mir-34c	unconfirmed	hsa-let-7e	dbdemc;miR2Disease
hsa-mir-34b	dbdemc	hsa-mir-7	dbdemc
hsa-mir-183	dbdemc	hsa-mir-223	dbdemc
hsa-mir-132	dbdemc	hsa-mir-106a	dbdemc;miR2Disease
hsa-mir-214	dbdemc	hsa-mir-205	dbdemc
hsa-mir-182	dbdemc	hsa-mir-222	dbdemc
hsa-mir-31	unconfirmed	hsa-mir-335	dbdemc
hsa-mir-133a	dbdemc	hsa-mir-27a	dbdemc
hsa-mir-212	dbdemc	hsa-mir-181c	dbdemc
hsa-mir-141	dbdemc	hsa-mir-224	dbdemc
hsa-mir-142	unconfirmed	hsa-mir-27b	dbdemc
hsa-mir-192	dbdemc	hsa-mir-30a	dbdemc
hsa-mir-429	unconfirmed	hsa-mir-370	unconfirmed
hsa-mir-451a	unconfirmed	hsa-mir-1	dbdemc
hsa-mir-106b	dbdemc	hsa-let-7g	dbdemc

The first column records top 1-25 related miRNAs. The second column records the top 26-50 related miRNAs.

Prostate neoplasms is a malignant tumor which originates in the epithelial cells of prostate [64]. Factors that increase the risk of prostate neoplasms include older age, a family history of the disease, race and a diet high in processed meat, red meat or milk products or low in certain vegetables [65]. Up to now, lots of miRNAs have been discovered to be associated with prostate neoplasms. For instance, the proto-oncogene ERG is a target of miRNA-145 in prostate cancer [66]. MCMDA predicts the top 10 and top 50 potential miRNAs which are associated with prostate neoplasms. As a consequence, 9 of the top 10 and 45 of the top 50 predicted miRNAs were confirmed in the dbDEMC and miR2Disease database (See Table 4).

**Table 4:** Prediction of the top 50 predicted miRNAs associated with prostate neoplasms based on known associations in HMDD database

miRNA	Evidence	miRNA	Evidence
hsa-mir-146a	miR2Disease	hsa-mir-150	dbdemc
hsa-mir-122	unconfirmed	hsa-mir-126	dbdemc;miR2Disease
hsa-mir-155	dbdemc	hsa-mir-195	dbdemc;miR2Disease
hsa-mir-21	dbdemc;miR2Disease	hsa-mir-29c	dbdemc
hsa-mir-34a	dbdemc;miR2Disease	hsa-mir-223	dbdemc;miR2Disease
hsa-mir-16	dbdemc;miR2Disease	hsa-mir-143	dbdemc;miR2Disease
hsa-mir-221	dbdemc;miR2Disease	hsa-mir-23a	dbdemc;miR2Disease
hsa-mir-29a	dbdemc	hsa-let-7a	dbdemc;miR2Disease
hsa-mir-133a	dbdemc	hsa-mir-200b	unconfirmed
hsa-mir-29b	dbdemc;miR2Disease	hsa-mir-214	dbdemc;miR2Disease
hsa-mir-15a	dbdemc;miR2Disease	hsa-mir-148a	miR2Disease
hsa-mir-26a	dbdemc;miR2Disease	hsa-mir-106b	dbdemc
hsa-mir-222	dbdemc;miR2Disease	hsa-mir-34c	dbdemc
hsa-mir-199a	dbdemc;miR2Disease	hsa-mir-142	unconfirmed
hsa-mir-1	dbdemc	hsa-mir-31	dbdemc;miR2Disease
hsa-mir-20a	miR2Disease	hsa-mir-141	miR2Disease
hsa-mir-17	miR2Disease	hsa-mir-182	dbdemc;miR2Disease
hsa-mir-15b	dbdemc	hsa-mir-200a	dbdemc
hsa-mir-19a	dbdemc	hsa-mir-101	dbdemc;miR2Disease
hsa-mir-19b	dbdemc;miR2Disease	hsa-let-7c	dbdemc;miR2Disease
hsa-mir-206	dbdemc	hsa-mir-192	dbdemc
hsa-mir-30b	dbdemc;miR2Disease	hsa-mir-181a	dbdemc;miR2Disease
hsa-mir-18a	unconfirmed	hsa-mir-9	dbdemc
hsa-mir-196a	dbdemc	hsa-mir-34b	dbdemc
hsa-mir-92a	unconfirmed	hsa-mir-133b	dbdemc

The first column records top 1-25 related miRNAs. The second column records the top 26-50 related miRNAs.

The result of case studies on the four aforementioned human diseases illustrates that MCMDA achieves excellent prediction performance. Moreover, we prioritized the potential miRNAs associated with all the human diseases in HMDD database (See Supplementary Table 1). We hope that the predictions of MCMDA can be verified in future scientific researches.

## DISCUSSION

Nowadays, researchers propose several computational methods to predict the potential associations between miRNAs and diseases because computational models could select the most promising miRNAs related to human diseases and are less expensive than the traditional experimental methods. In order to predict potential miRNA-disease associations, we developed a computational model of MCMDA by analyzing the known miRNA-disease associations and implementing the matrix completion algorithm to get the association score of each miRNA-disease pair. MCMDA obtained excellent prediction performances based on LOOCV and 5-fold cross validation. In addition, the predicted miRNAs associated with four important human diseases: colon neoplasms, kidney neoplasms, lymphoma and prostate neoplasms, were verified by the experimental literatures in dbDEMC and miR2Disease database. The results from cross validation and case studies indicated that MCMDA was effective in predicting potential miRNA-disease associations although it only depends on known miRNA-disease associations.

The reasons why MCMDA achieved excellent performances are as follows. Firstly, MCMDA predicts the miRNA-disease associations by using the matrix completion algorithm based on the observation that the miRNA-disease matrix is low-rank. MCMDA fills the candidate samples without known associations with 0 and then iteratively updates them with the predictive scores. Besides, MCMDA is based on the known miRNA-disease associations in HMDD database. Plenty of known associations guarantee the efficiency of the predictions in MCMDA. Finally, negative associations which are required in some previous models are not needed in MCMDA.

Yet, there still exist several limitations in MCMDA. Firstly, MCMDA method is based on the known miRNA-disease associations, which means it cannot predict the potential miRNAs associated with the new diseases without any known related miRNAs and potential diseases associated with new miRNAs. Besides, there is no powerful method to find the optimal parameters for MCMDA. Finally, the current miRNA-disease associations are insufficient. To be specific, there are merely 5430 known miRNA-disease associations within the possible exploration spaces of 495 miRNAs and 383 diseases. The more known associations are confirmed in the future, the more accurate MCMDA model can become.

## MATERIALS AND METHODS

### Human miRNA-disease associations

The known miRNA-disease associations were downloaded from HMDD v2.0 database [67] which consisted of 5430 known miRNA-disease associations, 495 miRNAs, and 383 diseases. We furthermore constructed an adjacency matrix M to represent known miRNA-disease associations. For instance, if miRNA m(i) is reported to be associated with disease d(j) in the database, the value of M(i,j) is 1 and otherwise 0. Ω denotes the set of all the known associations in matrix M which means (i,j)Ω if m(i) is associated with d(j). $n_{m}$ represents the number of miRNAs in HMDD database and $n_{d}$ represents the number of diseases.

### MCMDA

**Figure 2:**
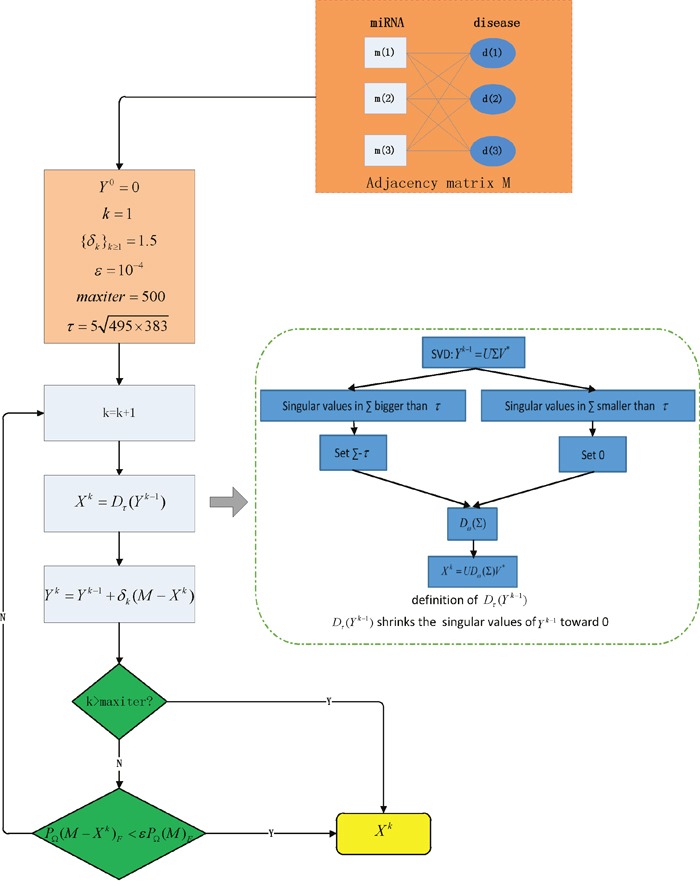
Flowchart of MCMDA model to predict the potential miRNA-disease associations based on the known associations in HMDD database

We developed MCMDA based on the known miRNA-disease associations in HMDD database to predict the potential associations (See Figure 2). MCMDA uses the singular value thresholding (SVT) algorithm to accomplish the matrix completion procedure. First, the miRNA-disease association matrix M was obtained according to known miRNA-disease associations. Here, all the known associations between miRNAs and diseases in HMDD database are used as training samples.

The matrix completion algorithm is iterative and a $n_{m}\times n_{d}$ prediction matrix $X^{k}$ (k denotes the iteration times) can be obtained in each iteration. When MCMDA ends, the matrix $X^{n}$ (n denotes the ultimate iteration times) is obtained which records the scores of all the possible miRNA-disease pairs. To ensure that the scores of known associations in $X^{n}$ are close to those in M, the following optimization problem needs to be solved.

$$\underset{X}{\min}f_{\tau }(X)$$

$$s.t.P_{\Omega }(X)=P_{\Omega }(M)$$ (1)

where X is a $n_{m}\times n_{d}$ candidate solution matrix with scores of all the unknown miRNA-disease samples, $P_{\Omega }$ is the orthogonal projector onto the span of matrices vanishing outside of Ω so that the (i,j) th component of $P_{\Omega }(X)$ is equal to X(i,j) if (i,j)∈Ω or zero otherwise. $f_{\tau }(X)$ is a nonlinear function of X which can be written as the following form.

$$\underset{X}{\min}\tau {\left\|X\right\|}_{*}+\frac{1}{2}{\left\|X\right\|}_{F}^{2}$$

$$s.t.P_{\Omega }(X)=P_{\Omega }(M)$$ (2)

where ${\left\|X\right\|}_{*}$ is the nuclear form of the matrix X which is the sum of the singular values of X, ${\left\|X\right\|}_{F}$ denotes the Frobernius form of X which is $\sqrt{\sum_{i=1}^{n_{m}}\sum_{j=1}^{n_{d}}X{\left(i,j\right)}^{2}}$, τ is athresholding which will be introduced later.

According to [68], problem (2) can be optimized using the Lagrangian multiplier method. Specifically, we introduce a Lagrangian multiplier Y and get the Lagrangian function as below:

$$L(X,Y)=f_{\tau }(X)+<Y,P_{\Omega }(M)-P_{\Omega }(X)>$$ (3)

The singular value decomposition (SVD) of matrix X with rank r, which represents the number of singular values of matrix X, is needed in matrix completion algorithm.

$$X=U\Sigma V*,\;\Sigma =diag({\left\{{\text{σ}}_{i}\right\}}_{i\leq i\leq r})$$ (4)

where U and V are $n_{m}\times r$ and $r\times n_{d}$ matrices. $\Sigma =diag({\left\{\sigma_{i}\right\}}_{1\leq i\leq r})$ means that Σ is a r×r diagonal matrix with positive singular values ${\left\{\sigma_{i}\right\}}_{1\leq i\leq r}$ on its main diagonal. For τ≥0, we introduce an operator $D_{\tau }$ defined as follows:

$$D_{\tau }(X)=UD_{\omega }(\Sigma )V*,\;D_{\omega }(\Sigma )=diag({\left\{\sigma_{i}-\tau \right)}_{+})$$ (5)

where ${\left\{\sigma_{i}-\tau \right\}}_{+}$ is the positive part of $\left\{\sigma_{i}-\tau \right\}$. In other words, ${\left\{\sigma_{i}-\tau \right\}}_{+}$ is equal to $\sigma_{i}-\tau$ if $\sigma_{i}-\tau \geq 0$ or 0 otherwise and it effectively shrinks the singular values of X toward 0. The value of τ is $5\sqrt{n_{m}\times n_{d}}$ according to the previous research of matrix completion algorithm [69].

There are two key steps which are special instances of Uzawa’s algorithm [70] to find a saddle point of (3) in each iteration. We introduce $\left\{Y^{0},Y^{1},...,Y^{n-1},Y^{n}\right\}$ which are a series of $n_{m}\times n_{d}$ matrices to record the intermediate scores of matrices $\left\{X^{1}..X^{n}\right\}$. First, update X with Y:

$$X^{k}=D_{\tau }(Y^{k-1})$$ (6)

Then, update Y with X:

$$Y^{k}=Y^{k-1}+\delta_{k}(M-X^{k})$$ (7)

where $Y^{0}$ is a zero matrix [71] and ${\left\{\delta_{k}\right\}}_{k\geq 1}$ is the step size. It is usually thought that the iteration can converge to an unique solution when 0<δ<2 [72], specifically, we empirically set the value of ${\left\{\delta_{k}\right\}}_{k\geq 1}=1.5$ according to the excellent performance in previous model [73]. MCMDA applies K.K.T conditions as the stopping criteria which are checked in each iteration to makes sure the scores of the known associations in the prediction matrix are close enough to the original matrix M:

$$P_{\Omega }{\left(M-X^{k}\right)}_{F}<\varepsilon P_{\Omega }{\left(M\right)}_{F}$$ (8)

where ε is a stopping tolerance, the value is $10^{-4}$ since it proved to be appropriate in restricting the iteration times in previous algorithm [71]. If the stopping criteria is met, MCMDA stops iteration immediately and the ultimate matrix $X^{n}$ is obtained. Finally, a parameter *maxiter* is set which restricts the max iteration times and avoids the infinite loop. Specifically, *maxiter* is set 500 to ensure that the ultimate matrix has reliable predicted scores. Based on the method mentioned above, the ultimate matrix $X^{n}$ is obtained by above calculation process which can be utilized to predict the potential miRNA-disease associations.

## SUPPLEMENTARY MATERIALS FIGURES AND TABLES




