# Supplementary material for: MCMDA: Matrix completion for MiRNA-disease association prediction
Source: Oncotarget. 2017 Feb 3;8(13):21187–99. doi: 10.18632/oncotarget.15061 (PMC5400576; doi:10.18632/oncotarget.15061)
Supplement: Supplementary file 1 [file oncotarget-08-21187-s001.pdf]

MCMDA: Matrix completion for MiRNA-disease association prediction

SUPPLEMENTARY TABLE

Supplementary Table 1: We applied MCMDA to prioritize all the candidate miRNA-disease pairs based on all the known miRNA-disease associations in HMDD database as training samples. This prediction result is published for further experimental validation and research

See Supplementary File 1
